# Accuracy of older adults in judging self-generated elbow torques during multi-joint isometric tasks

**DOI:** 10.1038/s41598-020-69470-5

**Published:** 2020-08-03

**Authors:** Ninghe M. Cai, Julius P. A. Dewald, Netta Gurari

**Affiliations:** 1 Department of Physical Therapy and Human Movement Sciences, Northwestern University, Chicago, IL 60611 USA; 2 Department of Biomedical Engineering, Northwestern University, Chicago, IL 60611 USA

**Keywords:** Perception, Sensory processing

## Abstract

Successful execution of daily activities requires accurate perception of the torques one generates about multiple joints. Even so, previous studies are mostly limited to an individual’s perception when torques are generated about a single joint. Consequently, this study investigates how accurately individuals judge torques at their arm during a multi-joint task. The accuracy of fifteen right-hand dominant participants (age: 60 ± 10 years) in matching isometric elbow torques, within the same arm, was quantified during single- and/or multi-joint tasks. Participants generated and matched elbow torques when the shoulder was: (1) not abducted (single-to-single-joint), (2) abducted (multi-to-multi-joint), and (3) abducted and then not abducted (multi-to-single-joint). The constant error for the multi-to-single-joint condition (dominant: 6.9 ± 5.9 Nm, non-dominant: 6.0 ± 5.5 Nm) was greater than that for the single-to-single-joint condition (dominant: 2.7 ± 3.1 Nm, non-dominant: 3.4 ± 2.8 Nm) (p < 0.001) and multi-to-multi-joint condition (dominant: 3.0 ± 2.8 Nm, non-dominant: 3.9 ± 2.7 Nm) (p < 0.001). The constant error for the multi-to-multi-joint condition did not significantly differ from that of the single-to-single-joint condition (p $=$ 0.780). Findings indicate that in older adults the perception of a self-generated torque during a 2-degree-of-freedom (DOF), multi-joint task is largely influenced by the motor commands associated with the 2-DOF task and is not specific to the DOF at each joint.

## Introduction

To carry out an intended movement, such as drinking from a cup, an individual must not only correctly generate, but also accurately perceive their self-generated torques^1^. Activities of daily living often require coordinated production of torques in multiple degrees-of-freedom (DOFs) across multiple joints at the upper-extremity. Even so, our understanding of how torques are perceived is largely limited to tasks requiring activation about one joint in a single DOF, or at the fingers^2–5^. As such, there is a need to understand an individual’s perception of torques during tasks that are more complicated and generalizable to the real-world. Consequently, this study investigates how accurately an individual can judge a torque at their arm during a 2-DOF, multi-joint task.

Literature suggests two possible sources of signals that contribute to the perception of torques^6,7^. One source is the afferent sensory signals arising from the periphery, namely from mechanoreceptors in the muscles, tendons, joint capsules, and skin^8–10^. The other source is the centrally generated signals related to the descending motor commands^11–14^. Previous studies demonstrated that individuals with a large fiber sensory neuropathy, who lack feedback from proprioceptors, can accurately match self-generated torques between arms^8,15^. Researchers have also manipulated the relationship between the efferent signal and motor output in individuals without neurological impairments by fatiguing muscles of or using a neuromuscular block to one of the arms^3,16–18^. While the goal of participants was to match the magnitude of the torques that they generated between arms, participants matched the level of muscle activation. Additionally, the relationship between descending motor commands and the perception of force was demonstrated when individuals were instructed to match a force across different muscle groups ^19^. The study found that participants matched the relative muscle activation rather than the absolute force. Although it remains an area of contention^3,20^, results from such studies generally support the idea that the central signals largely influence an individual’s perception of a torque^21–23^.

A term used to describe the centrally generated signals that give rise to the perception of torque is the sense of effort^7^. For a task involving a single DOF about a single joint, the sense of effort, which is related to the descending motor commands, is quantified as the torque that one generates in relation to the maximum torque that can voluntarily be produced^3,18^. Recent studies used motor activation patterns and kinematic measures as correlates of descending motor commands to demonstrate the influence of central signals on perceiving an external force^24,25^ and heaviness^26^ during multi-DOF tasks. However, how central signals contribute to one’s perception of a self-generated torque during a multi-DOF, multi-joint task remains unknown.

In this study, we investigated how accurately and precisely older adults could judge their self-generated torques during a multi-DOF, multi-joint task. Specifically, participants matched elbow flexion torques within a single arm during three different testing conditions. Given the controversial results on whether arm dominance affects perceptual accuracy^4,27–30^, we assessed both the dominant and non-dominant arm of participants. For the first condition, participants both generated and matched a reference elbow torque during a 1-DOF, single-joint task. This condition provided baseline perception for the 1-DOF task. For the second condition, participants both generated and matched a reference elbow torque during a 2-DOF, multi-joint task. This condition identified baseline perception for the 2-DOF task. For the third condition, participants generated a reference elbow flexion torque during the 2-DOF, multi-joint task and then matched this reference torque during the 1-DOF, single-joint task. This third condition is a novel testing paradigm that permitted us to use a protocol involving a single arm to probe at the contribution of the centrally-generated signals to the perception of a torque. Based on existing literature, we predicted that, similar to during a 1-DOF, single-joint task, the sense of effort is the main contributor to an individual’s perception of a torque during a multi-DOF, multi-joint task. Furthermore, we predicted that the sense of effort is related to the motor command associated with the 2-DOF task and not specific to each DOF. This prediction is based on the idea that the generation of a multi-DOF, multi-joint task is organized at a task-specific level and not at a level associated with an individual DOF^31^. Consequently, we predicted that the sense of effort associated with generating an elbow flexion torque during a 2-DOF, multi-joint task would be greater than the sense of effort associated with generating the same magnitude of elbow flexion torque during a 1-DOF, single-joint task. More specifically, we expected that the sense of effort would be greater if a person were to abduct to 40% of their maximum voluntary torque (MVT) at their shoulder and flex to 25% of their MVT at the elbow than if the person were only to flex to 25% of their MVT at their elbow. In turn, we hypothesized that torque-matching errors would be: (1) similar for our testing conditions when the DOFs were identical for the generated and matched reference torque, and (2) greater for the testing condition when the reference torque was generated during a 2-DOF, multi-joint task and matched during a 1-DOF, single-joint task.

## Methods

### Participants

This study was approved by the Northwestern University Institutional Review Board (STU00209165), and all experimental protocols were conducted according to the standards set by the Declaration of Helsinki. Fifteen individuals without neurological impairments [8 males, 7 females; age (mean ± SD): 60 ± 10 years] provided informed written consent prior to their participation. Inclusion criteria for all participants were: (1) no major injuries to either arm, (2) right-hand dominance as determined by the Edinburgh Handedness Inventory^32^, (3) an age older than 40, and (4) ability to understand and complete the experimental tasks.

### Experimental setup

**Figure 1:**
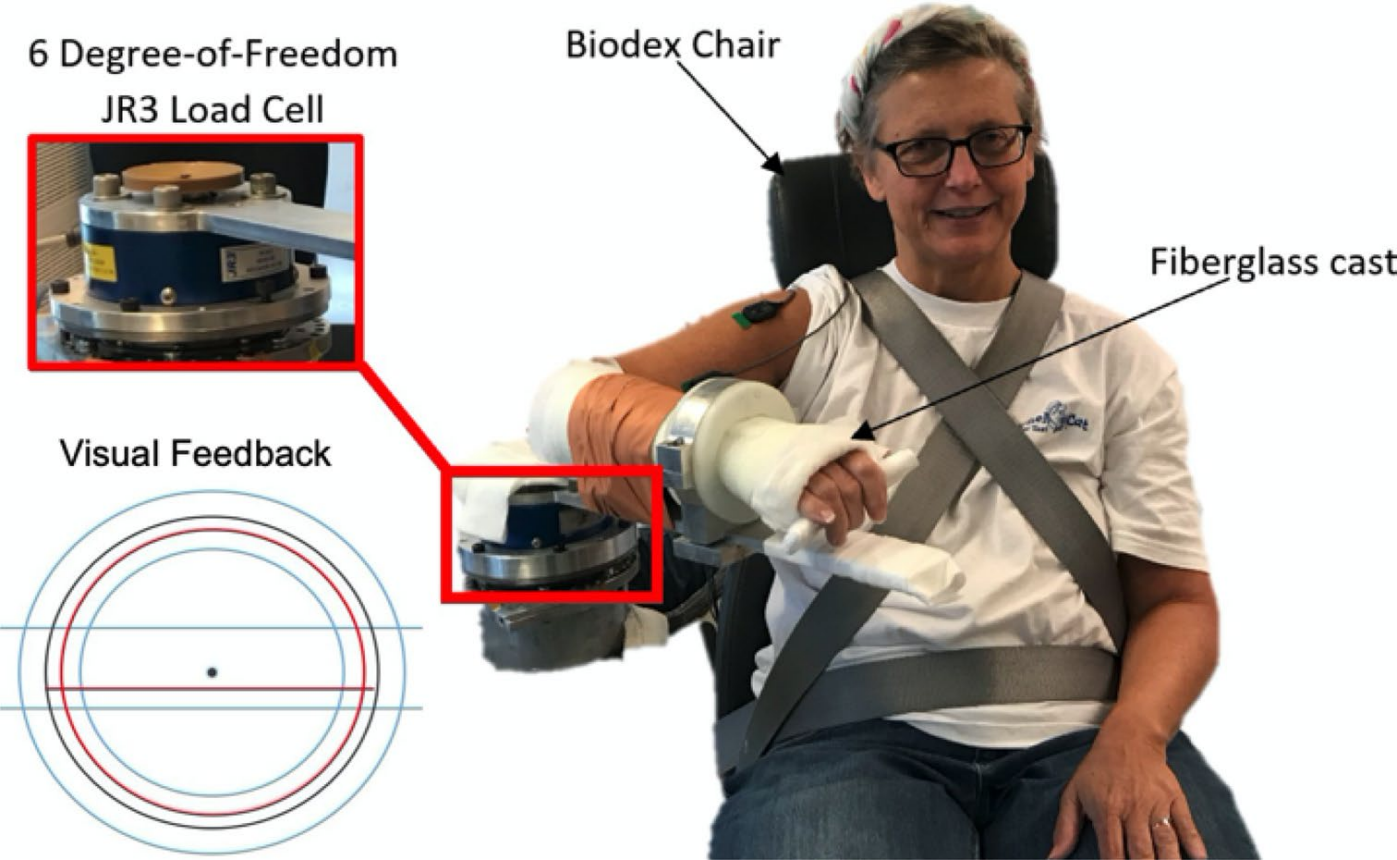
Isometric Experimental Setup. (Right) An example of a participant in the experimental setup affixed to the mechatronic device via a fiberglass cast. (Top, Left) A multi-axis load cell quantified the torques the participant applied about their testing elbow and shoulder. (Bottom, Left) Visual feedback providing information about the testing arm’s applied elbow flexion torque (red circle), target elbow flexion torque (black circle), allowable range of applied elbow flexion torques (blue inner and outer circles), applied shoulder abduction torque (red horizontal line), and allowable range of applied shoulder abduction torques (blue upper and lower horizontal lines). The individual shown in this figure provided written informed consent to publish this photo.

The experiment, as shown in Fig. 1, was conducted with participants seated in a Biodex chair (System 3 ProTM; Shirley, NY, USA). Seatbelts were used to restrict torso and waist movement. Participants were positioned so that their testing upper limb was situated in 85$^{\circ }$ shoulder abduction, 45$^{\circ }$ shoulder flexion, and 90$^{\circ }$ elbow flexion. The testing forearm was rigidly affixed to a mechatronic device, and, in turn, a 6-DOF load cell (JR3, Model: 45E15A 1000N; Woodland, CA, 88 USA) via a fiberglass cast. Visual feedback was provided to participants on a 42-inch monitor (Panasonic TH-42PH9; Osaka, Japan). Additionally, speakers played automated audio cues to instruct participants which actions to execute throughout the experiment.

### Experimental protocol

Participants were requested to not exercise the day before and of testing to avoid muscle fatigue. All testing procedures stated below were conducted first in one arm and then repeated in the other. The order of the arm first tested was randomized across participants.

To begin, participants generated their maximum isometric voluntary torque (MVT) with their testing arm. The MVT about their elbow joint in flexion, MTV$_{\mathrm{EF}}$, and about their shoulder joint in abduction, MVT$_{\mathrm{SAB}}$, was quantified. Next, we confirmed that participants could generate and hold 25% MVT$_{\mathrm{EF}}$ and 40% MVT$_{\mathrm{SAB}}$ for 4 s, independently and simultaneously.

The accuracy and precision of participants in judging their elbow torque during 1-DOF, single-joint and 2-DOF, multi-joint isometric tasks were then determined using three conditions (see “Torque-matching conditions” for details). For each of the three torque-matching conditions, participants began by performing two practice trials to gain familiarity with the methods. Then, participants performed eight testing trials, which were used in the data analyses. Participants did not receive feedback on their torque-matching accuracy. Presentation order of the torque-matching conditions was randomized across participants using a latin square design.

**Figure 2:**
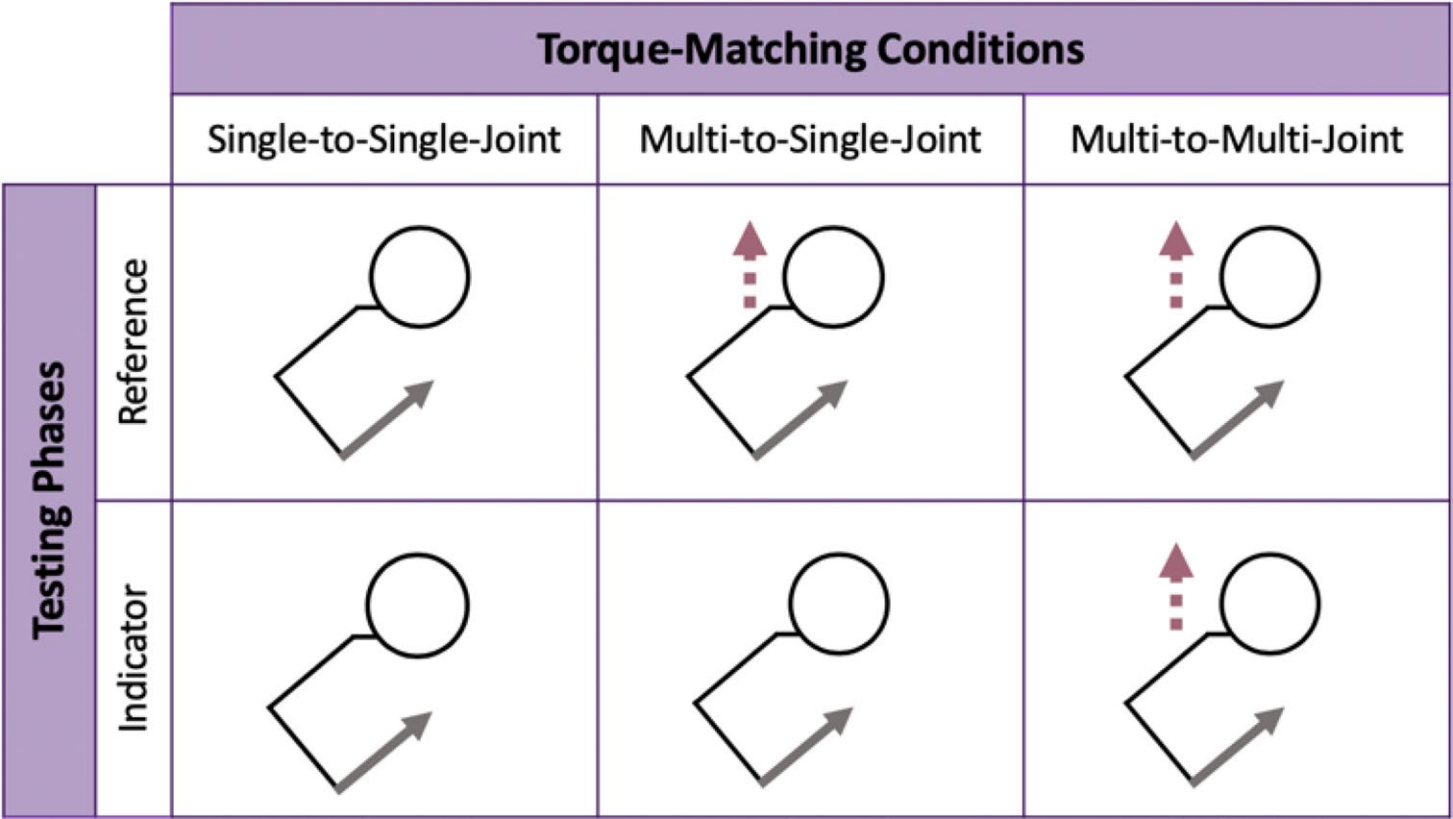
Summary of the Three Torque-Matching Conditions. The pictures depicted in the table are schematic drawings, providing a top-down view of the participant’s head, shoulder, and arm. The purple dashed arrows represent shoulder abduction, and the gray solid arrows represent elbow flexion. Participants matched the elbow flexion torque for all torque-matching conditions.

### Torque-matching conditions

Participants completed three isometric torque-matching conditions with each arm (Fig. 2). During the single-to-single-joint condition, participants generated and matched a 1-DOF flexion torque about their elbow. During the multi-to-multi-joint condition, participants generated and matched the elbow flexion torque during a 2-DOF, multi-joint task by simultaneously abducting at their shoulder. During the multi-to-single-joint condition, participants produced the reference elbow flexion torque while abducting at the shoulder during a 2-DOF, multi-joint task and subsequently matched this elbow torque without abducting at the shoulder during a 1-DOF, single-joint task.

### Torque-matching trial

**Figure 3:**
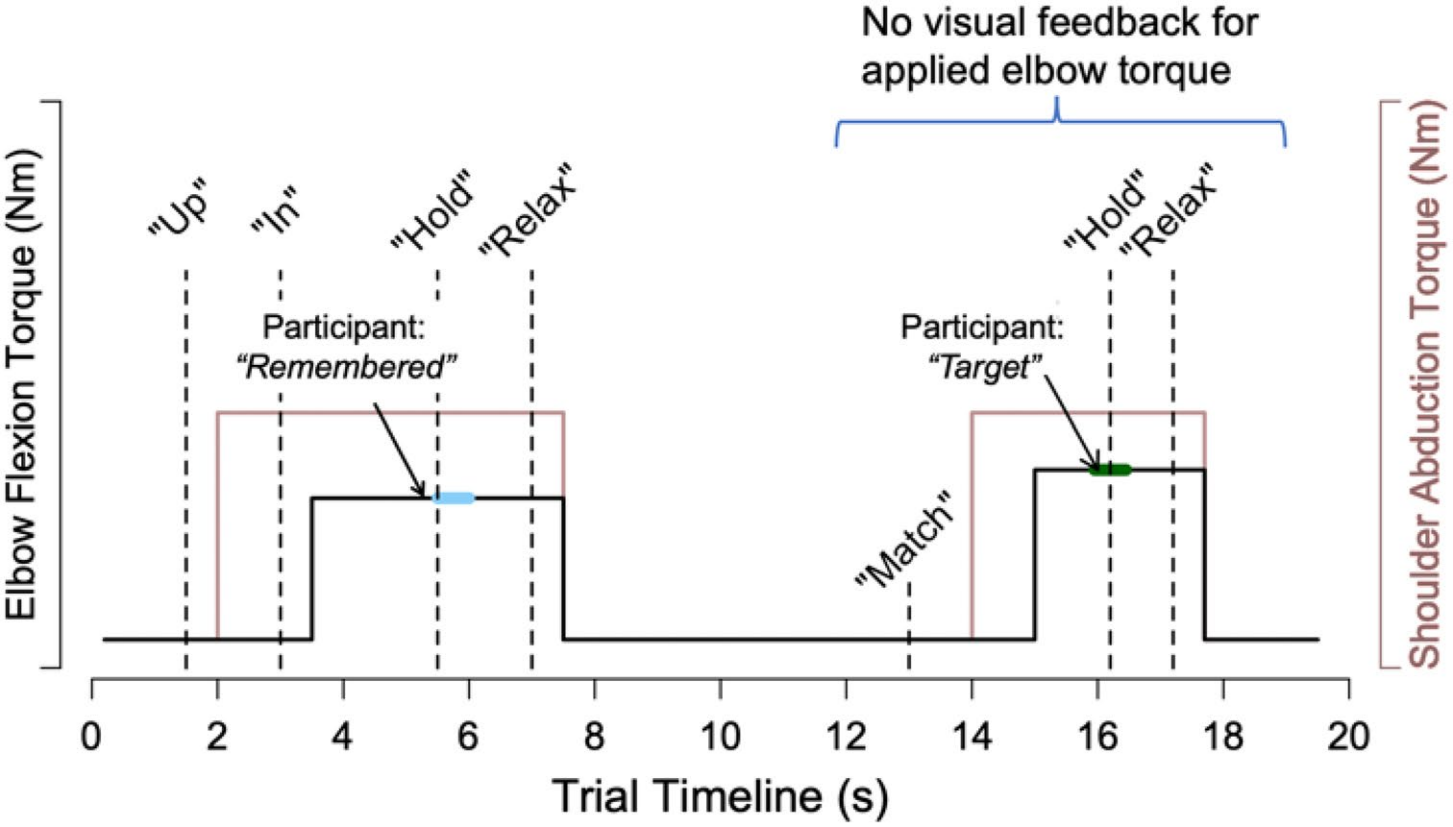
Schematic Trial Timeline for the Multi-to-Multi-Joint Matching Condition. Automated audio cues are indicated in italicized quotes. The bold blue and green lines indicate the segment of torque data that was averaged to be the reference elbow torque, $\tau _{\mathrm {reference}}$, and matching elbow torque, $\tau _{\mathrm {matching}}$, respectively.

During every trial, participants were required to match a flexion torque generated about their elbow. The sequence of events occurring during a trial is represented in Fig. 3 for the multi-to-multi-joint torque-matching condition. The target torque for elbow flexion was 25% MVT$_{\mathrm{EF}}$. For conditions involving the additional DOF at the shoulder (i.e., multi-joint task), the desired torque for shoulder abduction was 40% MVT$_{\mathrm{SAB}}$. The acceptable range of applied torques in elbow flexion and shoulder abduction was a minimum of 80% and a maximum of 120% of the target elbow and desired shoulder torque, respectively.

#### Visual feedback

Participants received visual feedback on their self-generated torques about the elbow and shoulder when generating the reference elbow flexion torque. When matching the reference elbow torque, participants only received visual feedback about their self-generated shoulder torque. The target elbow torque was visually depicted on the monitor as a stationary black circle, and the acceptable range of elbow torques that could be generated as the area between the inner and outer blue circles. The allowable range of the desired shoulder torques was depicted as the area between the blue upper and lower horizontal lines (Fig. 1 (Bottom, Left)). The elbow flexion torque generated by participants was shown on the screen as a red circle whose diameter changed corresponding to its magnitude, and the shoulder abduction torque as a red horizontal line whose height changed corresponding to its magnitude. To encourage that the visual feedback was easy for participants to use, the elbow flexion and shoulder abduction torques were filtered using a 9th-order Butterworth low-pass filter with a cutoff frequency of 5Hz prior to being displayed on the monitor. This filtering allowed for a smoother visual rendering of the participants’ dynamic circles and horizontal lines without a noticeable time lag.

#### Reference phase

The trial began with the target elbow torque visually depicted on the monitor. For the single-to-single-joint condition, participants began with the audio cue, “in”, instructing them to flex about their elbow to the target elbow flexion torque; participants were instructed to not abduct at their shoulder. For the multi-to-multi-joint and multi-to-single-joint conditions, after the trial started, participants were first instructed by the audio cue, “up”, to abduct at their shoulder. The trial continued when the shoulder abduction torque was held for 2 s within the acceptable range, i.e., the red horizontal line was situated between the two blue horizontal lines. Following, participants were cued by the audio cue, “in”, to flex about their elbow to the target elbow flexion torque. Across all three conditions, participants maintained the desired shoulder abduction torque and target elbow flexion torque and indicated to the experimenter that they remembered the target elbow torque. Subsequently, the experimenter pressed a key on the software to indicate that the elbow torque was being remembered. Following, an audio cue, “hold”, played to encourage participants to maintain the reference torque for two additional seconds. Participants then relaxed for 6 s to encourage that muscle activity subsided prior to the participants matching the reference torque^4^.

#### Matching phase

The start of the matching phase was signaled by the audio cue, “match”. During this phase, participants were required to match the torque generated about their elbow in the reference phase without receiving visual feedback about this torque. For the single-to-single-joint and multi-to-single-joint torque-matching conditions, participants did not abduct at their shoulder. For the multi-to-multi-joint torque-matching condition, participants abducted to the desired torque at their shoulder by relying on visual feedback. Subsequently, participants generated an elbow flexion torque and stated aloud to the experimenter “target” when the reference torque was perceived as matched. The experimenter pressed a key on the keyboard to indicate that the elbow torques were matched. Then, participants were encouraged to “hold” this matched torque for one additional second. Following, “relax” played, marking the end of the trial. To encourage quiescent muscle activity and avoid muscle fatigue, participants briefly activated their antagonist muscles and relaxed for 20s before beginning the next trial^33^.

### Data collection

Torques generated by the participants about their testing elbow and shoulder were estimated based on the forces and moments collected from a 6-DOF load cell^34^. Specifically, Jacobian transformation matrices with the required kinematic measurements were used to transform the load cell measurements to quantify the participants’ elbow and shoulder torques. The software updated at 4kHz, and the raw trial-related torque data were stored for post hoc analyses at 1kHz.

### Data analyses

#### Exclusion of trials

Based on visual inspection, we removed trials in which participants failed to hold their elbow and/or shoulder torque for one second after indicating “match” during the matching phase. Additionally, we excluded trials when participants abducted to more than 20% of their MVT$_{\mathrm{SAB}}$ at the shoulder if, during that phase of the trial, participants were only required to generate a 1-DOF torque about their elbow (i.e., no shoulder abduction). Finally, for each torque-matching condition, we excluded participants if fewer than three trials qualified for the data analyses.

#### Data extraction for the reference phase and matching phase

For each torque-matching trial, we extracted a 0.5s segment of data from the reference phase and matching phase (see Fig. 3 for a visual depiction). The reference segment was chosen as the 0.5s period after the audio cue, “hold”, played in response to participants stating that they remembered the reference torque. The matching segment was chosen as the data from 0.25s before and after “hold” played in the matching phase in response to participants stating that the reference torque was matched. Subsequent data analyses were performed based on the torque data collected during these two segments.

#### Control of elbow torques

For each condition, we quantified the participants’ control of their elbow flexion torques using the coefficient of variation of torques (CVT). CVT was determined by first identifying for each torque-matching trial, *i*, the variability of the elbow torques generated during the reference phase. That is, CVT$_{i}$ was quantified as the standard deviation normalized to the mean of the measured elbow torques during the reference segment of each trial. Then, CVT was calculated as the average CVT$_{i}$ across the eight testing trials for each torque-matching condition. As such, for the single-to-single-joint condition, CVT reflects the variability of the reference elbow torque during a 1-DOF, single-joint task since participants generated the reference torque without abducting about their shoulder. For the multi-to-single-joint and multi-to-multi-joint conditions, because participants generated the reference elbow torque while simultaneously abducting at their shoulder, CVT reflects the variability of the reference elbow torque during a 2-DOF, multi-joint task. The greater the CVT, the more variability participants had when maintaining the reference torque.

**Table 1:** Participant Information.

Participant	Age (years)/Gender	Dom Arm MVT$_{\mathrm{EF}}$ (Nm)	Non-Dom Arm MVT$_{\mathrm{EF}}$ (Nm)	Dom Arm MVT$_{\mathrm{SAB}}$ (Nm)	Non-Dom Arm MVT$_{\mathrm{SAB}}$ (Nm)
Control 1	56/M	65.4	70.8	45.4	57.0
Control 2	65/F	42.2	40.0	55.6	34.0
Control 3	64/M	74.0	80.5	95.0	91.8
Control 4	50/F	45.8	39.8	43.0	33.5
Control 5	56/F	42.0	40.5	42.7	43.2
Control 6	58/F	32.6	30.1	43.4	35.4
Control 7	57/M	72.4	80.6	113.3	114.4
Control 8	48/M	66.2	81.5	78.3	81.1
Control 9	86/F	29.8	28.0	32.3	33.5
Control 10	57/F	41.1	41.5	48.4	48.9
Control 11	70/M	72.4	68.0	71.9	55.9
Control 12	69/F	30.8	32.9	41.1	35.1
Control 13	45/M	45.0	40.3	48.8	40.0
Control 14	65/M	72.5	66.4	80.0	84.7
Control 15	57/M	60.1	52.7	61.4	55.2

This table summarizes relevant demographic and experimental information about each participant. M: male; F: female; Dom: dominant; Non-Dom: non-dominant; MVT$_{\mathrm{EF}}$: maximum voluntary torque in elbow flexion; MVT$_{\mathrm{SAB}}$: maximum voluntary torque in shoulder abduction.

#### Accuracy and precision in matching torques

For each condition, we quantified how accurately and precisely participants matched torques using the constant error and variable error, respectively. First, we identified for each torque-matching trial, *i*, a reference torque, $\tau _{\mathrm {reference,i}}$, and matching torque, $\tau _{\mathrm {matching,i}}$, as the average measured elbow torque during the reference and matching segments, respectively. The error in matching the reference torque for each trial was determined as $\tau _{\mathrm {matching,i}} - \tau _{\mathrm {reference,i}}$. A positive and negative error indicates that the reference torque was overestimated and underestimated, respectively. We visually inspected the errors across the eight testing trials for each condition to confirm that a decreasing or increasing trend of errors, which could be indicative of participants learning or fatiguing, was not observed. The constant error (CE) was calculated as the mean error across the eight testing trials. Participants, on average, overshot when matching torques if CE was greater than 0 and undershot if CE was less than 0. The variable error (VE) was calculated as the standard deviation of the errors across the eight testing trials. A large VE indicates that participants matched with greatly differing torques, while a VE close to zero indicates that participants consistently matched with the same torque.

### Statistical testing

We determined whether the control of the elbow torques (CVT), accuracy in matching the elbow torques (CE), and variability in matching the elbow torques (VE) significantly differed depending on the torque-matching condition and arm tested. We fit our data to linear mixed-effects models^35,36^; torque-matching condition, arm tested, and their interaction were treated as fixed effects, and participant was treated as a random effect. Analyses of variance were performed on the models using a hierarchical approach in which non-significant interaction terms were removed followed by non-significant main effect terms. Significantly differing levels were determined for significant main effects using post hoc pairwise comparisons. P-values were adjusted with the Tukey method to account for the multiple comparisons^37^.

### Ethics statement

This study was approved by the Northwestern University Institutional Review Board. Participants provided their written informed consent to participate in this study. Written informed consent was obtained from the individual for the publication of a potentially identifiable image in this article.

## Results

The experiment was designed to determine the accuracy and precision of older adults in judging their self-generated torques during a 2-DOF, multi-joint task. Prior to analyzing the torque-matching data, we removed 84 of the 720 trials based on our criteria for trial exclusion, as described in the methods section above. We removed eleven trials in the dominant arm from nine participants and nineteen trials in the non-dominant arm from nine participants due to failure to maintain the matching torque. In addition, 34 trials in the dominant arm from eight participants and fourteen trials in the non-dominant arm from four participants were removed because participants generated a substantial shoulder abduction torque when only a 1-DOF flexion torque about the elbow was required. As a result, for the multi-to-single-joint condition, Participant 8 and 13 each only had two trials remaining in the dominant arm. Due to the lack of trials, the data of Participant 8 and 13 in the dominant arm were not included in the analyses of the multi-to-single-joint condition. Participant 15 did not have any trials that qualified for data analyses in the non-dominant arm for the multi-to-single-joint condition and, as such, their data for this condition were not included in the data analyses. For the single-to-single-joint condition, Participant 13 did not have any trials that qualified for data analyses in the dominant arm and, likewise, their data for this condition were not included in the data analyses. Below we report the participants’ strength, control of their elbow torques, and accuracy and precision in matching elbow torques based on the remaining 636 trials analyzed.

**Table 2:** Summary of Outcome Measures when Matching Elbow Torques.

Arm		Single-to-single-joint	Multi-to-single-joint	Multi-to-multi-joint
Dominant	n	14	13	15
	$\mu _{\mathrm {torque}}$ (Nm)	13.1 ± 4.5	13.2 ± 4.5	13.2 ± 4.5
		(7.2, 19.5)	(7.4, 19.0)	(7.3, 18.8)
	CVT (%)	1.0 ± 0.2	1.2 ± 0.6	1.3 ± 0.5
		(0.7, 1.4)	(0.6, 2.7)	(0.5, 2.6)
	CE (Nm)	2.7 ± 3.1	7.6 ± 5.8	3.0 ± 2.8
		(–4.9, 7.3)	(–2.2, 18.3)	(–1.9, 7.8)
	VE (Nm)	1.9 ± 0.9	3.0 ± 1.6	2.1 ± 0.9
		(0.7, 2.9)	(1.0, 5.7)	(0.8, 3.9)
Non-Dominant	n	15	14	15
	$\mu _{\mathrm {torque}}$ (Nm)	13.1 ± 5.0	13.3 ± 4.7	13.2 ± 4.9
		(7.0, 20.1)	(7.6, 20.3)	(7.4, 20.3)
	CVT (%)	1.1 ± 0.4	1.3 ± 0.6	1.6 ± 0.6
		(0.7, 2.2)	(0.6, 2.8)	(0.8, 2.7)
	CE (Nm)	3.4 ± 2.8	6.2 ± 5.8	3.8 ± 2.7
		(–2.7, 8.7)	(–2.7, 17.5)	(0.0, 8.1)
	VE (Nm)	1.9 ± 0.9	3.0 ± 1.6	2.1 ± 0.9
		(0.9, 2.1)	(0.8, 5.8)	(1.0, 3.4)

Mean ± standard deviation and range (minimum, maximum) are reported across all participants for the mean magnitude of the measured reference torque ($\mu _{\mathrm {torque}}$), coefficient of variation of torques (CVT), constant error (CE), and variable error (VE) in the three conditions tested. n: number of participants included in the data analyses.

**Figure 4:**
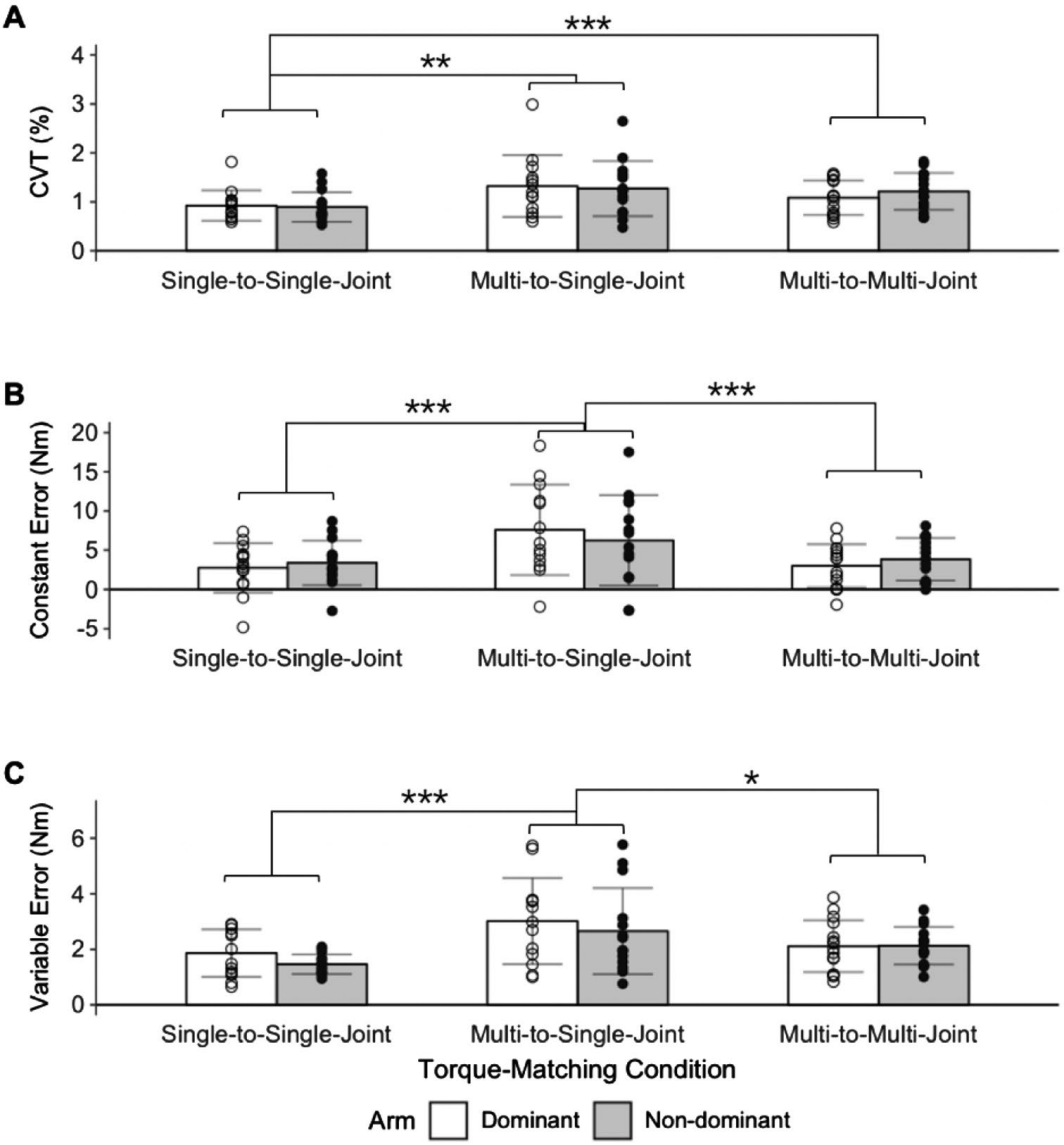
Outcome Measures when Matching Elbow Torques. Mean and standard deviation of participants’ **A** coefficient of variation of torques (CVT), **B** constant error, and **C** variable error. Each data point represents the outcome measure of one participant for each condition. Data for the dominant arm and non-dominant arm are identified by the white and gray bars, respectively (*p < 0.050; **p < 0.010; ***p < 0.001).

### Maximum voluntary torques

Table 1 summarizes the strength among participants in elbow flexion and shoulder abduction, as quantified by the MVTs. Across participants, the mean ± standard deviation MVT$_{\mathrm{EF}}$ generated by the dominant arm was 52.5 ± 16.5 Nm and by the non-dominant arm was 52.5 ± 18.8 Nm. The mean ± standard deviation MVT$_{\mathrm{SAB}}$ generated by the dominant arm was 59.0 ± 23.0 Nm and by the non-dominant arm was 55.5 ± 24.8 Nm. As a result, the target elbow torque of 25% MVT$_{\mathrm{EF}}$ corresponded to 13.1 ± 4.1 Nm for the dominant arm and 13.1 ± 4.7 Nm for the non-dominant arm. For the torque-matching conditions that included a second DOF, the desired shoulder abduction torque of 40% MVT$_{\mathrm{SAB}}$ corresponded to 23.6 ± 9.2 Nm for the dominant arm and 22.2 ± 9.9 Nm for the non-dominant arm.

### Control of elbow torques

The CVT during the reference phase for each torque-matching condition is reported in Table 2 and Fig. 4. The CVT was significantly affected by the torque-matching condition ($\hbox {F}_{(2, 69)}=8.420, \hbox {p}<0.001$), but not by the arm tested ($\hbox {F}_{(1, 68)}=0.225,~\hbox {p}=0.403$). The CVT was higher for conditions when participants held the reference torque with a shoulder abduction torque than without. Specifically, the CVT during the single-to-single-joint condition was less than that during the multi-to-single-joint ($\hbox {p}<0.001$) and multi-to-multi-joint ($\hbox {p}=0.003$) conditions, suggesting that the ability to steadily control elbow torques was negatively affected when participants abducted at their shoulder. The CVT did not significantly differ between the multi-to-single-joint and multi-to-multi-joint conditions ($\hbox {p}=0.168$) when participants held the reference elbow torque while abducting at their shoulder.

### Accuracy and precision in matching torques

The accuracy and precision of participants when matching the reference elbow torque under each torque-matching condition is summarized in Table 2 and Fig. 4.

CE was affected by the torque-matching condition ($\hbox {F}_{(2, 69)}=16.951, \hbox {p}<0.001$), but was not found to depend on the arm tested $(\hbox {F}_{(1, 68)}=0.177, \hbox {p}=0.675)$. The constant error of the multi-to-single-joint condition was larger than that of the single-to-single-joint $(\hbox {p}<0.001)$ and multi-to-multi-joint $(\hbox {p}<0.001)$ conditions. The constant error of the multi-to-multi-joint condition did not significantly differ from that of the single-to-single-joint condition $(\hbox {p}=0.780)$.

VE was affected by the torque-matching condition $(\hbox {F}_{(2, 69)}=12.582, \hbox {p}<0.001)$, but was not found to depend on the arm tested $(\hbox {F}_{(1, 68)}=1.318, \hbox {p}=0.255)$. The variability in torque-matching errors during the multi-to-single-joint condition was larger than that during the single-to-single-joint $(\hbox {p}<0.001)$ and multi-to-multi-joint $(\hbox {p}=0.031)$ conditions. The variable error of the multi-to-multi-joint condition did not differ from that of the single-to-single-joint condition $(\hbox {p}=0.203)$.

## Discussion

This work investigates how accurately older adults match elbow flexion torques within a single arm during single-joint (1-DOF task) and multi-joint (2-DOF) tasks. We showed that participants accurately matched elbow flexion torques within a single arm during single-joint (1-DOF task) and multi-joint (2-DOF) tasks. However, participants overestimated their self-generated torques when they referenced this elbow torque during the 2-DOF task and matched during the 1-DOF task. This study provides evidence that the perception of a self-generated torque in older adults during a multi-DOF, multi-joint task is largely influenced by the sense of effort, or the motor commands associated with the task. Below we discuss our findings, identify limitations of the current work, and indicate future research directions.

Given the inherent link between motor control and perception, we wanted to first present our results indicating whether a change in how torques are generated potentially influenced our perceptual results. Our results characterizing how well the reference torque could be controlled without and with a shoulder abduction load revealed that the variability in generating elbow torques was greater when participants abducted at their shoulder for both the dominant and non-dominant arm. This finding is aligned with previous studies on motor control that showed an increase in force variability when torques are generated about multiple DOFs^38–42^. In contrast, prior studies indicated that arm dominance affects the variability in postural control^27,43^ whereas we did not observe a significant impact of arm dominance on the control of reference torques. An earlier study also showed that an increased variability in the reference torque can negatively impact how accurately torques are perceived^44^. However, this adverse impact of increased variability in torque generation to the accuracy in matching torques is not apparent in our study. The torque-matching error for the dominant and non-dominant arm during the single-to-single-joint torque-matching condition did not significantly differ from that during the multi-to-multi-joint condition despite the increased variability in the reference elbow torque in the latter. Additionally, the variability in the reference torque was similar between the multi-to-single-joint and the multi-to-multi-joint conditions, yet the constant error significantly differed between the two conditions. As such, the difference in the constant error between conditions does not correspond to the difference observed in the variability in controlling one’s torques. Therefore, the errors in matching torques do not seem to be driven by changes in the motor control.

Next, we report our findings for the perceptual testing. The literature has not reached a consensus on whether arm dominance affects how accurately a torque is perceived. Several studies showed that force-matching errors could be affected by the handedness of the reference arm^27,28^, while others failed to observe such a difference^4,30^. In this study, we did not find a significant effect of the arm tested on the accuracy of perceiving elbow torques for any of the torque-matching conditions.

Data from the single-to-single-joint condition identify an individual’s baseline perception about their elbow when judging a sub-maximal torque generated in 1-DOF and one direction. It is worth noting that the magnitude of constant error and variable error reported for the single-to-single-joint condition in this study is consistent with our previous study utilizing a similar single-joint torque-matching protocol^4^. The mean positive constant error indicates that participants tended to overestimate their reference elbow flexion torque when matching within in arm. The mean variable error of less than 2 Nm in both arms suggests that participants consistently overestimated when matching the elbow flexion torque. The overestimation reflected by the positive constant error has been observed in previous studies when a participant matches a force to a reference force that was previously passively applied^45,46^. These earlier studies found that sensory perception for self-generated forces is attenuated (i.e., reafference attenuation), resulting in an overestimation when matching to a passively-applied force. However, our torque-matching condition involved self-generated torques for both the reference and matching phases. As such, the proposed mechanism of reafference attenuation does not address why our participants overestimated the torques when matching during the single-to-single-joint condition in one DOF. All the same, we conclude that baseline performance for matching a torque during the single-joint task has an error greater than zero. Future work can investigate the reason why torques are overestimated when both the reference torque and matching torque are self-generated. Errors for the remaining two conditions were compared to those of this single-to-single-joint condition to allow for a meaningful interpretation of the results.

Findings from the multi-to-multi-joint condition establish an individual’s perception when performing a 2-DOF, multi-joint task. We found that the constant error in the multi-to-multi-joint condition did not significantly differ from that in the single-to-single-joint condition (p>0.050). Differences in constant errors between the two conditions were less than 0.5 Nm. Likewise, variable error in the multi-to-multi-joint condition did not significantly differ from that in the single-to-single-joint condition (p>0.050). Therefore, we do not have reason to believe that activating about the shoulder influenced how accurately and precisely participants perceived their elbow torques. Results for this condition support our hypothesis that participants relied primarily on the sense of effort to perceive a torque during the 2-DOF, multi-joint task. Similar to the single-to-single-joint condition, the multi-to-multi-joint condition requires a comparable level of muscle activation in the matching phase to accurately match the reference torque generated in the reference phase. Given that the constant errors in these two conditions were similar, the level of activation in the reference phase may have been matched in the matching phase.

Generating an elbow flexion torque when abducting at the shoulder (2-DOF, multi-joint task) requires greater muscle activation at the arm than generating the same elbow flexion torque when relaxing at the shoulder (1-DOF, single-joint task). Consequently, we expected that for the multi-to-single-joint condition participants would overestimate the reference elbow flexion torque if they relied on the sense of effort. That is, the sense of effort would be large when generating the elbow torque while also abducting at the shoulder during the multi-joint task (reference phase), and the sense of effort would be comparatively small when only generating the elbow torque while resting at the shoulder during the single-joint task (matching phase). In turn, participants would match an elbow torque generated during the multi-joint task (reference phase) with a larger elbow torque than during the single-joint task (matching phase). As such, torque-matching errors for the multi-to-single-joint condition would be greater than for the single-to-single-joint and multi-to-multi-joint conditions. Our results supported this hypothesis by demonstrating that participants matched with greater constant errors in both the dominant and non-dominant arms during the multi-to-single-joint condition than the other two conditions. Additionally, participants matched the elbow flexion torque with significantly higher variability in the multi-to-single-joint condition, indicating a level of perceptual uncertainty^47^. Similar findings were observed by a previous study that examined the accuracy in the perception of forces during an action involving numerous effectors, specifically forces produced by multiple fingers^30^. Results from this study indicate that when individuals generated a force with four fingers in one hand and matched the force produced by one of the fingers with the corresponding finger in the other hand, they consistently overshot and matched with higher variability than if the reference and matching forces were generated by the same number of fingers. In the current study, the increase in constant error and variable error during the multi-to-single-joint condition suggests that participants cannot accurately isolate their judgement to only their elbow torque during a 2-DOF, multi-joint task involving shoulder abduction. We conclude that our participants likely matched the elbow flexion torque based on the sense of effort associated with the multi-joint task, which is not specific to each joint.

One limitation of this study is that we do not have a manner by which to quantify the sense of effort for our 2-DOF, multi-joint task. In the context of forces produced about a single joint in one DOF, literature indicates that the sense of effort is related to the size of the motor commands and can be reflected by the extent of the muscle activation^2,17^. As such, the sense of effort is often quantified for a 1-DOF, single-joint task as a ratio of the generated force to the maximum voluntary force^18,48^. However, research has yet to demonstrate how this relationship between motor commands and the sense of effort can be generalized to a task that relies on multiple DOFs and joints. One approach could be to use methods such as electromyography to quantify to what extent associated muscles are activated. Yet, this approach presents its own set of challenges given that many muscles, including the trunk, may be activated during tasks requiring shoulder abduction and elbow flexion. Hence, quantifying the sense of effort during a multi-DOF, multi-joint task remains an area of research for future work.

Another limitation is that the multi-joint task used in this study is isometric, meaning that limb position does not change. Even though our task more closely resembles a real-world action than an isometric single-joint task, our approach is still not necessarily a natural movement that one would produce in everyday life. Findings from this study are important for reporting on one’s perception at the upper limb during a task involving multiple DOFs and joints. However, the degree to which our results can be extended to the perception of torques generated during movements remains to-be-determined.

To conclude, findings from this study suggest that in an older adult population perception of a self-generated torque during a multi-DOF, multi-joint task is largely influenced by the sense of effort, or the motor commands associated with the task. It remains to-be-determined whether these findings are generalizable to a younger adult population as studies have shown aging to have an effect on proprioceptive signaling and motor control capabilities^6,38,49,50^. Future work plans to expand this line of research to quantify the impact of a change in the motor output about one joint on the perception of torque about a second joint during a 2-DOF task involving two joints. Furthermore, future work aims to address how accurately individuals with hemiparetic stroke perceive their self-generated torques during a multi-DOF, multi-joint isometric task. Current literature indicates that individuals with hemiparetic stroke have altered accuracy in estimating forces generated^5,51–53^ and are challenged with controlling independent joint movements due to abnormal muscle coactivation patterns in the paretic upper limb^54–58^. Therefore, future work will address the effect of changes in motor commands on the accuracy of judging self-generated torques during a multi-DOF, multi-joint task in individuals post-hemiparetic stroke.

## Data Availability

The raw data supporting the conclusions of this manuscript will be made available by the authors, without undue reservation, to any qualified researchers.
